# MoonProt: a database for proteins that are known to moonlight

**DOI:** 10.1093/nar/gku954

**Published:** 2014-10-16

**Authors:** Mathew Mani, Chang Chen, Vaishak Amblee, Haipeng Liu, Tanu Mathur, Grant Zwicke, Shadi Zabad, Bansi Patel, Jagravi Thakkar, Constance J. Jeffery

**Affiliations:** 1 Department of Bioengineering, University of Illinois at Chicago, Chicago, IL 60607, USA; 2 Department of Biological Sciences, MC567, University of Illinois at Chicago, 900 S. Ashland Ave., Chicago, IL 60607, USA; 3 Center for Pharmaceutical Biotechnology, College of Pharmacy, University of Illinois at Chicago, 900 S. Ashland Ave., Chicago, IL 60607, USA; 4 Illinois Institute of Technology, 3300 S Federal St, Chicago, IL 60616, USA

## Abstract

Moonlighting proteins comprise a class of multifunctional proteins in which a single polypeptide chain performs multiple biochemical functions that are not due to gene fusions, multiple RNA splice variants or pleiotropic effects. The known moonlighting proteins perform a variety of diverse functions in many different cell types and species, and information about their structures and functions is scattered in many publications. We have constructed the manually curated, searchable, internet-based MoonProt Database (http://www.moonlightingproteins.org) with information about the over 200 proteins that have been experimentally verified to be moonlighting proteins. The availability of this organized information provides a more complete picture of what is currently known about moonlighting proteins. The database will also aid researchers in other fields, including determining the functions of genes identified in genome sequencing projects, interpreting data from proteomics projects and annotating protein sequence and structural databases. In addition, information about the structures and functions of moonlighting proteins can be helpful in understanding how novel protein functional sites evolved on an ancient protein scaffold, which can also help in the design of proteins with novel functions.

## INTRODUCTION

It has been known for many years that some soluble enzymes have a second function as structural proteins in the lens of the eye (crystallins) (1,2) or in binding to DNA or RNA to regulate translation or transcription (3). These proteins belong to a subclass of multifunctional proteins called ‘moonlighting proteins’ (4), in which more than one biochemical or biophysical function is contained within one polypeptide chain. In this subclass of multifunctional proteins, the multiple functions are not due to gene fusions, multiple RNA splice variants or multiple proteolytic fragments. Moonlighting proteins do not include pleiotropic proteins, where a protein has multiple downstream cellular roles in different pathways or physiological processes that result from a single biochemical function of a protein. Moonlighting proteins also do not include families of homologous proteins, if the different functions are performed by different members of the protein family.

Over 200 proteins have been found to moonlight, and it is possible that many other proteins also have additional functions that have not yet been found. The known examples of moonlighting proteins include diverse types of proteins, including receptors, enzymes, transcription factors, adhesins and scaffolds [for reviews see (3–16)]. Moonlighting proteins are expressed in many cell types and function in many different biochemical pathways. They are found in mammals, yeast, bacteria, plants and other organisms. Different combinations of functions are observed, for example, a cytoplasmic enzymatic function and an extracellular cytokine function are found in phosphoglucose isomerase/autocrine motility factor (17–20).

Some moonlighting proteins can perform both functions simultaneously, but for others, the functions of a moonlighting protein can vary due to changes occurring within the cell. A moonlighting protein can adjust to these changes by having different functions in different cellular locations, in different cell types, by interacting with other proteins in various oligomeric states or when it senses a change in the cellular concentration of a ligand, substrate, cofactor or a product of an enzymatic reaction (4). Two proposed mechanisms/models (1,4) for the evolution of a moonlighting function make use of general features of protein structure and could apply to many protein types, potentially resulting in a large number of proteins that moonlight. The diverse examples of moonlighting proteins already identified, the methods by which one protein can moonlight, the two proposed models for the evolution of moonlighting functions and the potential benefits moonlighting proteins might provide through coordinating cellular activities, suggest that moonlighting proteins are likely to be common.

The ability of a single protein to have multiple, sometimes unrelated, functions and to be involved in different multiprotein complexes or biochemical pathways can make more complicated predicting functions of the many genes identified through genome sequencing projects, interpreting results from proteomics experiments and annotating protein structure and sequence databases. In proteomics studies, the additional functions can result in complicated protein expression and interaction patterns and make it more difficult to determine if ‘false’ positives are truly false or perhaps instead due to a second function of a protein. Moonlighting proteins complicate the interpretation and annotation of genome sequences because sequence homologs can share all, none, one or some functions. For example, the delta 1 and delta 2 crystallins in the duck lens share 94% amino acid sequence activity, but only the delta 2 isoform retains arginosuccinate lyase catalytic activity (21–24). In systems biology, moonlighting adds another level to our understanding of the complex but organized cellular protein network. For example, a moonlighting protein can provide a means of communication and coordination between the many pathways within a cell or a mechanism to switch between pathways, helping the cell to respond to changes in its environment. A moonlighting protein can also be involved in communication and coordination between different cell types within an organism.

Information about the structures and functions of moonlighting proteins can be helpful in studies of protein evolution, for example, in understanding how novel protein functional sites evolved on an ancient protein scaffold. This information can also be useful for the design of proteins with novel functions because the structures and functions of moonlighting proteins can serve as a guide of how to add new functional site to the fold of a stable protein. In moonlighting proteins nature has created new functions on old protein scaffolds many times, and for some proteins it is clear which function was the original functional site on a protein structure and which functional site evolved later.

For the reasons listed above, it would be useful to have a way to identify moonlighting proteins, but there is currently no straightforward method to identify which proteins have moonlighting functions or for determining if a protein of interest moonlights. The lack of an identified common physical feature has prevented a means of quickly identifying other moonlighting proteins. In addition, characterization of a novel protein generally involves finding a function for a protein, but it does not necessarily include a search for all possible additional functions of a protein. For most moonlighting proteins a second function has been found by serendipity.

Information about moonlighting proteins is scattered in many publications. There is currently some confusion in the literature because some authors have used the term ‘moonlighting’ when the publication is actually describing a protein family in which different members have different functions, or a protein that has the same function but in two different locations (two biological roles but not two biochemical functions), or a protein that is another type of multifunctional protein. The presence of a protein in an unexpected location can suggest that the protein has a second function, but additional evidence that the protein truly performs two different biochemical functions in the two locations is needed before concluding that a protein moonlights.

The MoonProt Database provides a centralized, searchable, web-based database for knowledge about the known moonlighting proteins. It contains information about the sequences, structures and functions for over 200 moonlighting proteins for which multiple biochemical functions have been experimentally verified within one protein. The MoonProt database is also the first database that is strictly devoted to moonlighting proteins and not other types of multifunctional proteins. It differs from another recent resource that includes multifunctional or ‘multitasking’ proteins that have multiple functions due to gene fusions, proteins that have a pleiotropic effect on multiple pathways and/or ‘multifunctional proteins’ for which the two functions are not exhibited by a single protein but instead by different protein products within a multiprotein complex (25).

In contrast, for the MoonProt Database, we have carefully selected only proteins that have two different biochemical or biophysical functions, such as being a crystallin in the lens of the eye in addition to being an enzyme with catalytic activity. This specific requirement adds to the value of the MoonProt database and is particularly important for some studies of protein structure and function, protein evolution, protein function prediction and the design of proteins with novel proteins.

The MoonProt Database was created in order to be a resource for researchers and for educational purposes. The availability of this organized, curated information provides a more complete picture of what is currently known about moonlighting proteins. This repository of information integrates information scattered across the literature and provides links to other databases. It helps avoid the need of searching through hundreds of journal articles. It will also serve as a resource for the future development of methods to identify other moonlighting proteins. The database will be important for many labs that are working on determining the functions of genes identified in the genome sequencing projects, interpreting data from proteomics projects, studying the evolution of new functions on proteins, designing proteins with novel functions and annotating protein sequence and structural databases. Also, researchers in other fields, such as cell biology or genetics, will be able to look up the proteins they are studying and see if they are known to moonlight.

## MATERIALS AND METHODS

### Selection of moonlighting proteins included in the database

The goal of creating the MoonProt Database is to bring together information about the experimentally validated moonlighting proteins. We manually collected information from peer-reviewed journals with an emphasis on finding references describing experimental evidence of multiple biochemical and/or biophysical functions within one polypeptide chain. Published biochemical, mutagenic or other evidence to support the presence of multiple functions was required and was critically reviewed by the Principle Investigator (PI) before the protein was accepted for inclusion in the MoonProt Database. Ideally, independence of both functions has been shown by demonstrating that inactivation of one function (i.e. through mutagenesis) does not inactivate the second function, and vice versa.

Proteins were not included if they belong to a different class of multifunctional proteins, for example, if the ‘multiple functions’ are due to gene fusions, different RNA splice variants, the same function in two different locations, pleiotropic affects on multiple pathways or multiple physiological processes or a family of proteins in which the different functions are performed by different proteins. We also have not included proteins for which the ‘multiple functions’ are simply different aspects of the same function (i.e. ‘membrane protein’ and ‘transmembrane channel’). In this version 1.0 of the database, we also include a list of ‘potentially moonlighting proteins’, for which a second function is only suggested by the results of gene knockout or other genetics experiments, in the FAQs page to illustrate examples of additional proteins that might also moonlight.

### Information included about individual proteins

Information about each protein was manually curated from published journal articles and online resources (Protein Data Bank (PDB), Uniprot, etc.). In some cases, the curators communicated with the labs that are experts in studying the individual proteins for assistance in finding some of the information (for example, finding the correct amino acid sequence, identifying which species was used in the experiments or checking the accuracy in our descriptions of the biochemical functions). The protein-specific entries include a description of each biochemical or biophysical function and a list of citations for evidence of that function. Alternative names are included because many moonlighting proteins have multiple names relating to their multiple functions. Including the alternative names aids in finding a protein of interest in a text search of the database. The specific species in which each protein has two or more functions was identified; it is especially important that the correct species and amino acid sequence is identified for the moonlighting protein and not for a homolog that might or might not have both functions. UniProtKB (26) or Pubmed [http://www.ncbi.nlm.nih.gov/pubmed/] resources were used to identify the correct amino acid sequences. The sequences are included in FASTA format. Basic Local Alignment Search Tool [http://blast.ncbi.nlm.nih.gov/Blast.cgi] was used to identify structures in the PDB (27) that correspond to the amino acid sequence, if available. Protein structures are not listed if they belong to a homolog from a different species. Gene Ontology (GO) terms (28) were identified from the UniProtKB Database (26) in order to illustrate the different types of proteins annotated in the MoonProt Database. UniProtKB (26) entry IDs and Enzyme Commission (EC) numbers are included for easy connection to external resources. When available, information is included about the cellular location in which the protein exhibits each function.

Members of the Jeffery laboratory will be in charge of updating the database and verifying the multiple functions of additional moonlighting proteins that are continuing to be identified. It is also anticipated that more information about the proteins on the list will become available, including new protein structures, additional functions, new information about functional sites on the protein structures, additional evidence of each function (i.e. additional biochemical or biophysical experiments), as well as the corresponding references.

## DATABASE ARCHITECTURE AND WEB INTERFACE

The MoonProt Database is based on a MySQL database for storing data (http://www.mysql.com/) and uses PHP 5.2.11 (http://www.php.net/). The web pages are written in Hypertext Markup Language and provide a user-friendly interface for locating and retrieving information from the database.

**Figure 1.**
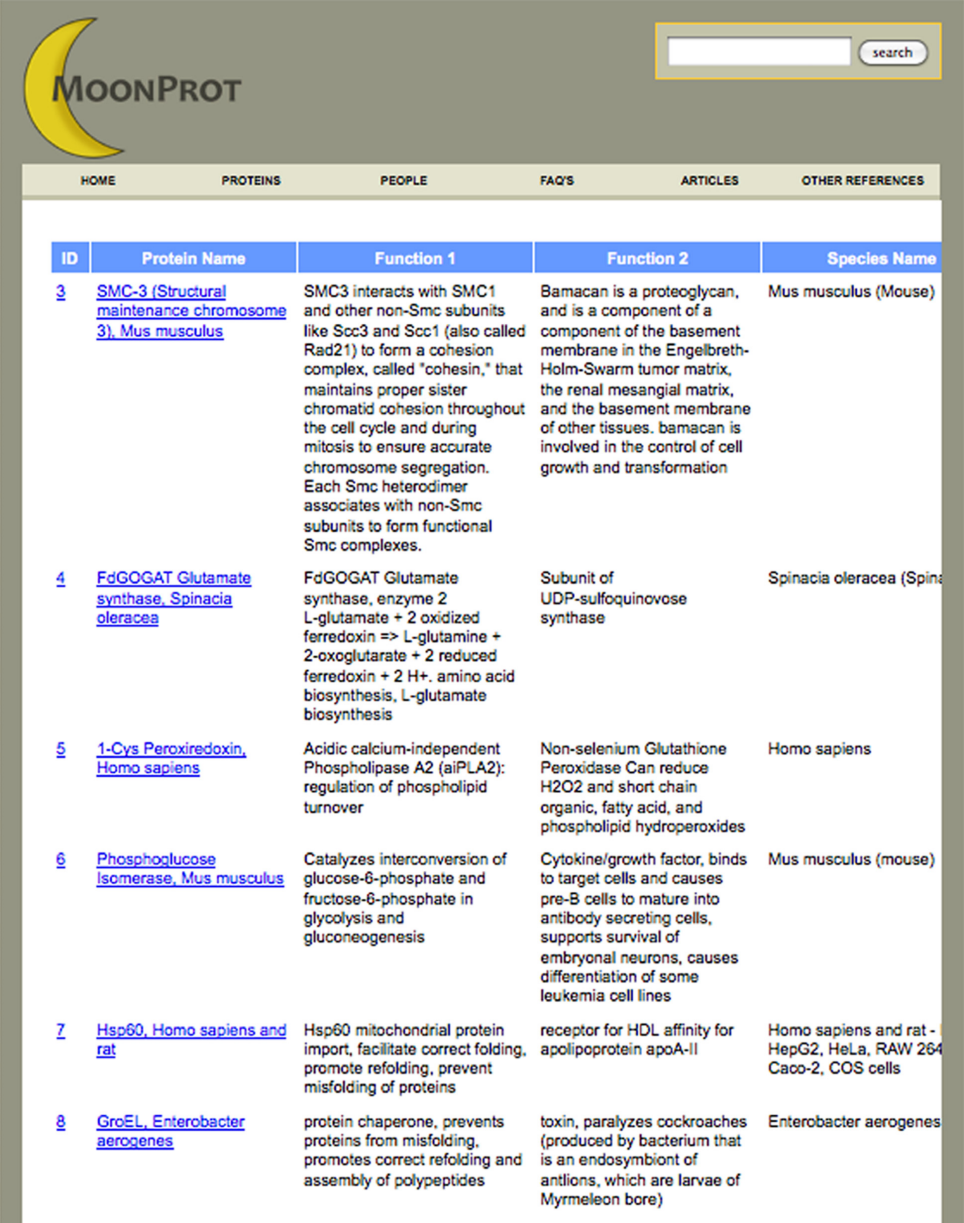
The Proteins Page enables users to browse a list of all the proteins annotated in the MoonProt Database. Version 1.0 of the database contains over 200 moonlighting proteins.

**Figure 2.**
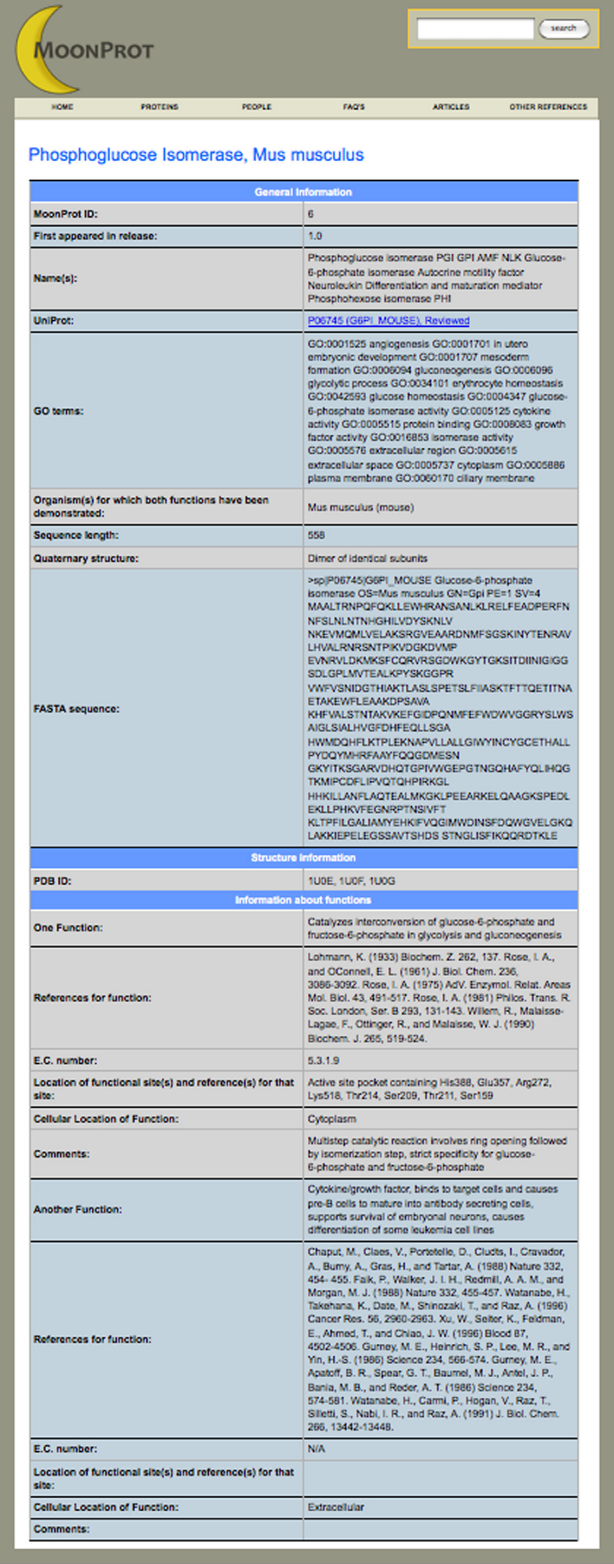
The Protein Details page brings up annotation for a selected protein that was manually curated to include alternative names of the protein, information about its biochemical and/or biophysical functions, citations describing experimental evidence of the functions, the specific species in which the protein has two or more functions, the amino acid sequence in FASTA format, PDB IDs of any available structures, GO terms from the UniProtKB resource (23), EC numbers for enzyme functions and other information.

The moonlighting protein database includes a total of seven types of pages: Home, Proteins, People, FAQ's, Articles, Other References and a Protein Details page that can be used to bring up the annotated information about each moonlighting protein in the database. The website features a prominent header that makes it easy to explore and discover relevant information. In the header, there is a navigation bar that provides an easy way to navigate between the main sections of the website, as well as a text search box that makes it easy for the user to query and retrieve specific information from the database. The Home page provides a summary about moonlighting proteins and a dynamic illustration of several moonlighting proteins and their functions. The Proteins page (Figure 1) provides a list of all the proteins included in the database. From the Proteins page, the user can select a protein and bring up the annotated information for that protein in the Protein Details page (Figure 2). The annotated information for each protein contains the names of the protein, a UniProt accession number, GO terms from the UniProt database, the species of organism for which both functions have been demonstrated to be in one protein, the length of the amino acid sequence, the amino acid sequence in FASTA format, PDB IDs for any available protein structures in the PDB, descriptions of at least two functions, references for experiments demonstrating the functions and EC numbers for enzymes. Additional information is included for some proteins, including cellular location where that function is observed, and information about quaternary structure. The FAQ's page includes frequently asked questions about moonlighting proteins and information about how to contact the database curators. The Articles page includes a list of review articles about moonlighting proteins. The Other References page includes references to the UniProtKB, PubMed and other resources used in the creation of the database.

## RESULTS AND CONCLUSIONS

The MoonProt Database brings together information about over 200 moonlighting proteins in a centralized, web-based resource. It includes proteins from species throughout the evolutionary tree, archaea, bacteria and eukaryotes (mammals, plants, reptiles, insects, worms, protozoa, etc.). Receptors, enzymes, transcription factors, adhesins, chaperones and scaffolds, and many other types of proteins are included.

Having information about confirmed moonlighting proteins together in one place and separate from other types of multifunctional proteins (i.e. gene fusions) will facilitate studies of their sequences, structures and functions. The proteins in the database can also serve as a test set for testing algorithms and programs to predict protein functions in general and for predicting which other proteins might moonlight. For example, because of the different physical arrangement of the functional sites in moonlighting proteins versus gene fusion proteins, a program may score well in predicting from sequence or structure the two functions of a gene fusion protein but not identify the multiple functions of a moonlighting protein. The information in the database is already being used in tests of protein function prediction methods.

The database can also serve as a set of examples of proteins and data (protein sequences, functions and in many cases structures) to aid in developing hypotheses about which characteristics, motifs or other features might be shared by moonlighting proteins, or some subtypes of moonlighting proteins, that could potentially be used in developing new methods to identify moonlighting proteins. In addition, by comparing the list of proteins with the results of proteomics studies that compare protein–protein interactions, protein expression levels or protein cellular locations, an interaction, expression or location pattern might be identified for proteins that moonlight that can be used to predict which other proteins in those studies might also moonlight.

In proteomics studies, the MoonProt Database can also be useful in another way. Because moonlighting proteins are often found in more than one cellular location or expressed in more than one cell type or interact with multiple binding partners, they might help explain unexpected protein localization, protein expression or protein–protein interaction results. Proteins identified in the proteomics experiments can be compared to the list of known moonlighting proteins to see if the presence of a second function can help explain the unexpected results.

The MoonProt Database may also be valuable to researchers who identify a protein of interest in cell biology, genetics or other areas of biology or medicine. Information about all the known functions of a protein might help in identifying the specific function, and the associated biochemical pathway or multifunctional complex, that might be involved in a physiological process or disease being studied.

Finally, the MoonProt Database includes links to the 3D structures of 115 moonlighting proteins in the PDB. These include proteins that have been adopted for a second function without significant changes to sequence or structure, proteins that have gained a second binding site during evolution, proteins that undergo conformational changes to switch between function and examples of other ways in which a protein can have two different functions. These structures, as well as the amino acid sequences for these and many other moonlighting proteins, can provide interesting examples for studying the evolution of new protein functions, and they can provide examples and potential guides for designing and regulating the functions of proteins with novel functions,

The Jeffery lab is continuing to review the literature to add additional moonlighting proteins for future updates of the database as well as to add additional information and references for the proteins included in version 1.0. We welcome suggestions of additional moonlighting proteins and information about those proteins from colleagues.

## AVAILABILITY

The MoonProt Database is freely available via a user-friendly graphical user interface at the web address www.moonlightingprotiens.org. The interface enables text search for a protein name, species, amino acid sequence or a UniProtKB or PDB identifier. The user can also browse a list of all the proteins in the database. The database is ‘read and search only’ by the public, but additional information about the known moonlighting proteins and suggestions of other proteins that might also be moonlighting are welcome and can be sent to the curators for possible inclusion in the database.
